# Solution Properties and *in Vitro* Anti-Tumor Activities of Polysaccharides from Longan Pulp 

**DOI:** 10.3390/molecules180911601

**Published:** 2013-09-18

**Authors:** Yang Yi, Fei Huang, Ming-Wei Zhang, Rui-Fen Zhang, Yuan-Yuan Deng, Zhen-Cheng Wei, Jing-Ren He

**Affiliations:** 1 Key Laboratory of Functional Food, Sericulture and Agri-Food Research Institute, Guangdong Academy of Agricultural Sciences, Guangzhou 510610, China; 2 College of Food Science and Engineering, Wuhan Polytechnic University, Wuhan 430023, China

**Keywords:** longan pulp, polysaccharide, solution property, conformation, anti-tumor activity

## Abstract

The solution properties of four fractions (LPI–IV) from crude longan pulp polysaccharides (LP3) were analyzed by size-exclusion chromatography combined with laser light scattering, viscometry, complex formation with Congo red, and atomic force microscopy. Their radii of gyration (<S^2^>_z_^1/2^) were 43.3, 62.6, 43.2 and 77.3 nm, exponents of <S^2^>_z_^1/2^ = *k M*_w_*^v^* were 0.04, 0.50, 0.52 and 0.02, and intrinsic viscosities ([*η*]) were 9.945, 25.38, 308.2 and 452.1 mL/g, respectively. Moreover, the dependence of [*η*] on *M*_w_ was established to be [*η*] = 5.3 × 10^−2^*M*_w_^0.61^ (mL/g). LPI had both a sphere-like conformation and a triple-helix structure, and LPII–IV existed as flexible chains. LP3, LPI, LPII and LPIII all exhibited direct inhibitory effects on A549, HeLa and HepG2 cells in a positive dose-dependent manner in the range of 50–400 µg/mL. The activities of LPIII, especially the inhibition of HepG2 cell proliferation, were stronger than those of others, which may be partly related to its flexible conformation. The present results support the cancer therapeutic potential of longan polysaccharides.

## 1. Introduction

Polysaccharides isolated from botanical sources (algae, lichens and higher plants) have broad bioactivities and generally do not cause significant side effects [1]. Polysaccharides are ideal drug candidates considering their excellent anti-tumor and/or immunomodulatory activities, which are closely related to their structural features [2,3,4]. For example, the immunomodulatory activities of polysaccharides from *Ganoderma lucidum* [5], *Aloe* [6] and *Chlorella pyrenoidosa* [7] importantly depend on their molecular masses. Commonly, the triple-helix structure contributes greatly to the anti-tumor and immunomodulatory activities of glucans [7,8,9,10,11]. Besides, polysaccharides from *Pleurotus tuber-regium* [12,13] and *Poria cocos* [14] all exist as sphere-like conformations and exhibit strong anti-tumor activities. Random coil polysaccharides from *Cordyceps militaris* could also strengthen the functional events mediated by activated macrophages, such as nitric oxide production and cytokine (IL-1β and TNF-α) expression [15]. As a result of these differences, the solution properties of polysaccharides, such as molecular mass and conformation, should be investigated to explore their potential structure-activity relationships.

Longan (*Dimocarpus longan* Lour.) is an attractive fruit, commercially distributed in subtropical areas. Its pulp has been traditionally used to promote blood metabolism, soothe nerves, relieve insomnia and prevent forgetfulness [16,17]. Recent studies have indicated that the beneficial functions of longan pulp are partly related to the immunomodulatory effects of its water-soluble polysaccharides [18,19]. In our previous work, the chemical structures of four fractions (LPI–IV) purified from crude longan pulp polysaccharides (LP3) have been analyzed, and their compositions, molecular masses, configurations and glycosidic linkages have been confirmed [20]. However, their aqueous solution properties, which are non-ignorable factors for exploring the molecular mechanisms of their bioactivities, still remain unclear. In addition, longan pulp polysaccharides have anti-tumor effects in S180 tumor mice, possibly via an immunomodulation mechanism, displaying the potential to be used as immunoadjuvants for the immunotherapy of cancer [18], but their direct inhibitory effects on tumor cells are unavailable. The present work aimed to investigate the aqueous solution properties of longan pulp polysaccharide fractions, including molecular size, intrinsic viscosity and conformation, and evaluate their *in vitro* anti-proliferation effects on A549, HeLa and HepG2 tumor cells.

## 2. Results and Discussion

### 2.1. Aqueous Solution Properties of Longan Polysaccharides

#### 2.1.1. Root-Mean-Square Radius of Gyration

**Figure 1:**
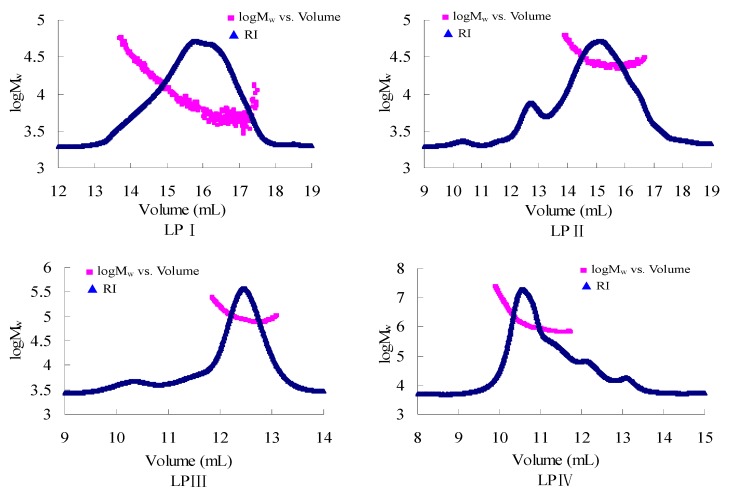
The size-exclusion chromatograms of longan polysaccharide fractions LPI–IV detected by a refractive index detector in water at 25 °C.

As the size-exclusion chromatograms shown in Figure 1 indicate, LPI, LPII and LPIV all exhibited a large overlapped-peak indicating a broad molecular mass distribution. LPIII, by contrast, showed a rather narrow peak with a polydispersity index close to 1. The *M*_w_ logarithm values of LPI–III did not decrease with increasing elution volume in the chromatograms, like those of *Rhizoma Panacis Japonici* polysaccharides, which implies the possibility of chain aggregation [21].

<S^2^>_z_^1/2^, which reflects how far from the centre of mass and how the mass of the polymer chain is concentrated can effectively describe the dimensions of polysaccharide chains. As seen in Table 1, LPIII had a larger *M*_w_, but a smaller <S^2^>_z_^1/2^, compared with LPI and LPII. Likewise, the characteristic of larger *M*_w_ with smaller <S^2^>_z_^1/2^ was also confirmed in *Rhizoma Panacis Japonici* polysaccharide RPS5 *vs*. RPS4 [21] and *Pleurotus tuber-regium* polysaccharide *vs*. its sulfated derivative [22]. It was thus indicated that the molecular chains of LPIII were relatively compact possibly because of the mechanism of aggregation.

**Table 1:** Parameters of <S^2^>_z_^1/2^ = *k M*_w_*^v^* and Huggins equation of longan polysaccharide fractions LPI–IV.

Polysaccharide	<S^2^>_z_^1/2^ (nm)	*v*	[*η*] (mL/g)	k'
LPI	43.3	0.04 ± 0.00	9.945	1.1879
LPII	62.6	0.50 ± 0.04	25.38	0.1951
LPIII	43.2	0.52 ± 0.03	308.2	0.0048
LPIV	77.3	0.02 ± 0.00	452.1	0.0047

**Figure 2:**
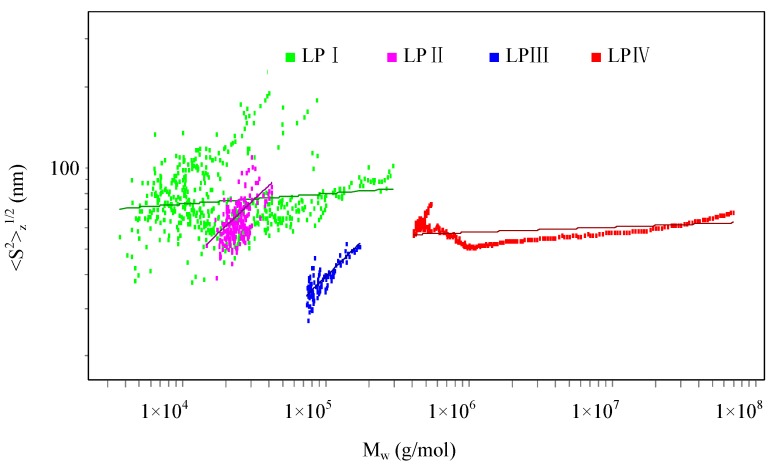
The double logarithmic plots of <S^2^>_z_^1/2^ *vs.* *M*_w_ of longan polysaccharide fractions LPI–IV.

Fractal dimension (*d*_f_) is a parameter describing the usually non-integer (fractal) power of mass increasing with radius for polymers in solution. The *d*_f_ value is defined as the inverse of the exponent *v* of equation <S^2^>_z_^1/2^ = *k M*_w_*^v^*, that is, *d*_f_ = 1/*v*. For a rigid rod-like polymer, its *v* value is 1.00. A linear polymer with Gaussian coil nature has a *v* value range of 0.50 to 0.60. Branching can decrease the *v* value with respect to its linear counterpart. A three-dimensional polymer with a homogeneous density has a *v* value of 0.33 [12,21,22]. The double logarithmic plots of <S^2^>_z_^1/2^ *vs.* *M*_w_ of LPI–IV are shown in Figure 2, and the *v* values are listed in Table 1. The *v* values of both LPII and LPIII were 0.5, characterizing fully swollen branched macromolecules in a thermodynamically good solvent [23], and their molecular chains might exist as random coils. The low *v* values of LPI and LPIV were close to those of *Pleurotus tuber-regium* polysaccharide TM2 and its carboxymethylated derivative, indicating the existence of globular conformations [22], which could be related to the high branching structure of the polysaccharides.

#### 2.1.2. Intrinsic Viscosity

**Figure 3:**
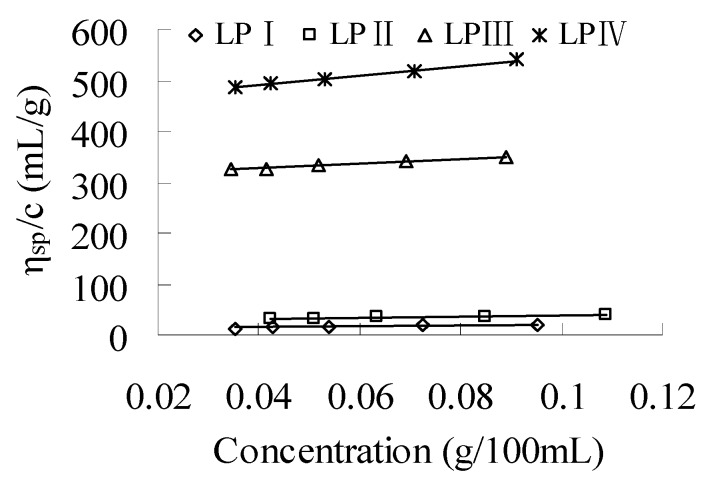
Huggins’ plot of longan polysaccharide fractions LPI–IV in water at 25 °C.

As shown in Figure 3, good linear relationships between the *η*_sp_/c and concentration of LPI–IV correspond to the normal solution behavior of polysaccharides, but not to polyelectrolyte behavior [21,24]. 

By extrapolating the linear plots of *η*_sp_/c *vs*. concentration to infinite dilution, the *M*_w_ dependent [*η*] of LPI–IV in water were obtained, as shown in Table 1. As a sensitive parameter for the extension of polymer chains, [*η*] could partly reflect the shape and volume of LPI–IV in water [21,25]. The [*η*] of LPIV was obviously higher than that of spherical polysaccharide from *Pleurotus tuber-regium* and lower than that of random coil polysaccharide from *Ganoderma tsugae* [12,26], implying the existence of semi-flexible chains. The Mark-Houwink equation of longan polysaccharides was further established to be: [*η*] = 5.3 × 10^−2^*M*_w_^0.61^ (mL/g). Generally, the α values of 0.5, 0.6–0.8 and >1 indicate that polymer molecules behave as dense spheres, flexible chains and elongated rods, respectively [12]. The structure of a polymer is theoretically a perfect hard sphere when its α value is 0 [27]. The experimental α value of 0.61 indicated that longan polysaccharides in the *M*_w_ range of 1.459 × 10^4^–5.282 × 10^6^ g/mol existed as flexible chains in water at 25 °C. Yomakawa-Fujii-Yoshizaki theory for the [*η*] of unperturbed wormlike cylinder was further adopted for the conformational characteristic of longan polysaccharides using the following Equations (1)–(3) [4,26,28,29]:

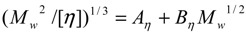
(1)

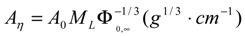
(2)

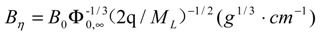
(3)
where *d* was the chain diameter (nm); Flory constant Φ_o,∞_ was 2.87 × 10^23^ when d_r_ ≤ 0.1; *N*_A_ was the Avogadro constant of 6.02 × 10^23^; and *υ* was the partial differential specific volume, for polysaccharide was 0.68 cm^3^/g. (*M*_w_^2^/[ŋ])^1/3^ *vs.* *M*_w_^1/2^ was plotted to establish Equation (1): (M_w_^2^/[ŋ])^1/3^ = 171.639 + 1.478M_w_^1/2^. The *A_ŋ_* value of 171.639 and the *B_ŋ_* value of 1.478 were substituted into Equation (5) to obtain the d_r_^2^/A_0_ value of 9.445 × 10^−3^, and further yield the d_r_ value of 0.096 and the A_0_ value of 0.972 via Equation (6). Finally, the values of *M_L_*, *q* and *d* were respectively calculated to be 1,164.120 (nm^−1^), 6.946 (nm) and 1.334 (nm) according to Equations (2)–(4): where *q* was the persistence length (nm); *M_L_* was the molar mass per unit contour length (nm^−1^); B_0_ was a constant with the value range of 1.05 to 1.08, and 1.065 was used in this paper; A_o_ was calculated by the following Equations (4)–(6):

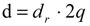
(4)

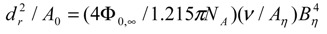
(5)

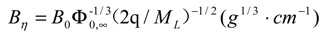
(6)


The parameters *M_L_* and *q* are important for characterizing the conformation and rigidity of molecular chain, and the greater values are, the more rigid is the chain. As reviewed by Yang *et al*. [4], commonly, the values of *M_L_* and *q* respectively range from 400–2,200 nm^−1^ and 3–200 nm. The *M_L_* and *q* values of longan polysaccharides were relatively close to those of semi-flexible curdlan (890 nm^−1^, 6.8 nm) [30] and semi-stiff sulfated glucan from *Poria cocos* (1060 nm^−1^, 13.1 nm) [31], larger than those of random-coil polysaccharides from *Pleurotus tuberregium* (408 nm^−1^, 3.1 nm) [32] and *Ganoderma tsugae* (832 nm^−1^, 4.2 nm) [26], and much smaller than those of triple helical lentinan (2,240 nm^−1^, 100 nm) [11] and single helical succinoglycan (1,500 nm^−1^, 50 nm) [33]. It was implied thus that longan polysaccharides in the *M*_w_ range of 1.459 × 10^4^–5.282 × 10^6^ g/mol existed as semi-flexible chains.

### 2.2. Helical Structures of Longan Polysaccharides

**Figure 4:**
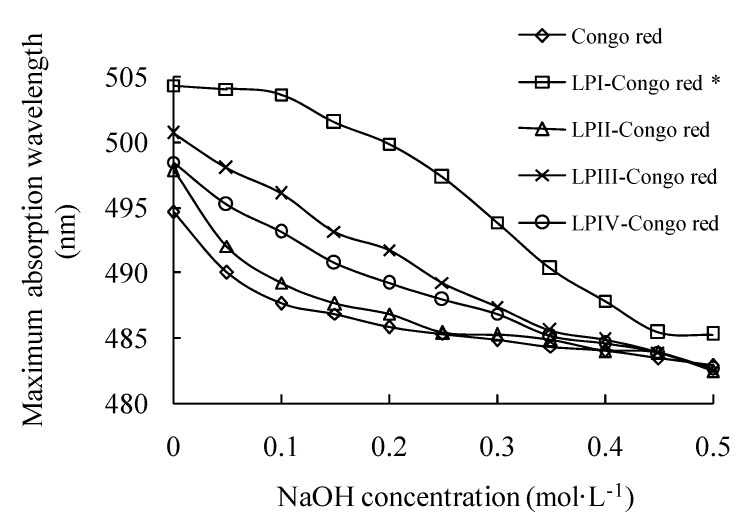
The maximum absorption wavelengths of longan polysaccharide-Congo red complexes at the NaOH concentration range of 0–0.5 mol/L; * represents the data obtained from our previous work [36].

As a colorant, Congo red can combine with helical polysaccharides, especially single-helical ones, resulting in a red shift of λ_max_ [34,35]. The λ_max_ of longan polysaccharide-Congo red complexes in the NaOH concentration range of 0–0.5 mol/L are shown in Figure 4. The triple-helix structure of LPI has been indicated by comparing with the control of curdlan. In detail, the nearly unaltered λ_max_ of LPI-Congo red complex from NaOH concentration 0 to 0.05mol/L responded to the depolymerization of triple-helix to single helix. Its subsequent decreases responded to the change from single helix to random coil [36]. In contrast, the λ_max_ of other complexes constantly decreased as that of Congo red control, and were obviously lower than that of LPI-Congo red complex at same NaOH concentration, indicating LPII–IV all had low-organized conformation without triple-helix structure [37].

### 2.3. Atomic Force Microscopy Images of Longan Polysaccharides

**Figure 5:**
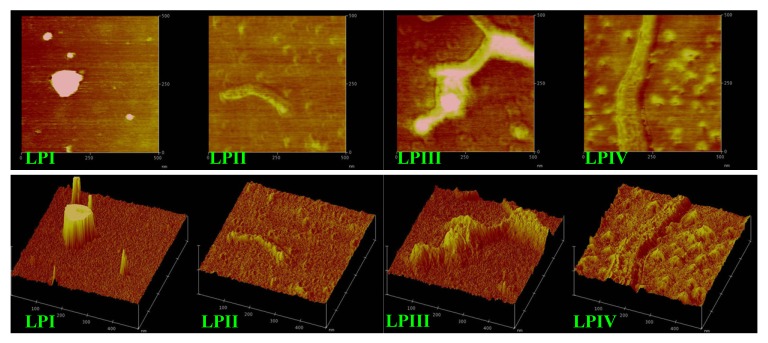
The atomic force microscope images (500 × 500 nm) of longan polysaccharide LPI–IV. 5 μL of sample (1 × 10^−3^ mg/mL) was air dried onto mica and imaged. The phase diagrams and corresponding three-dimension graphics were shown. The images of LPI were obtained from our previous work [36].

Atomic force microscope images of LPI–IV in distilled water are shown in Figure 5. The well-dispersed spherical particles of LPI in the diameter range of 20–120 nm were imaged clearly. 

Their sizes were close to those of *Rhizoma Panacis Japonici* polysaccharides (30–200 nm) [21]. LPII and LPIV existed as extended semi-flexible chains. LPIII was observed in a cross-linked network-like conformation, which might be related to intermolecular aggregation induced by the negative charge repulsion between the polysaccharide and mica. The polysaccharide chain width generally ranged from 0.1 to 1.0 nm. However, the widths observed were obviously larger than the theoretical value (1.334 nm) seen in the atomic force microscopy observation of konjac glucomannan [29]. A reasonable explanation for that was the broadened domino effect caused by the interaction between tiny needlepoint and different sections of molecular chain during the course of scanning.

### 2.4. Anti-Tumor Activities of Longan Polysaccharides

**Figure 6:**
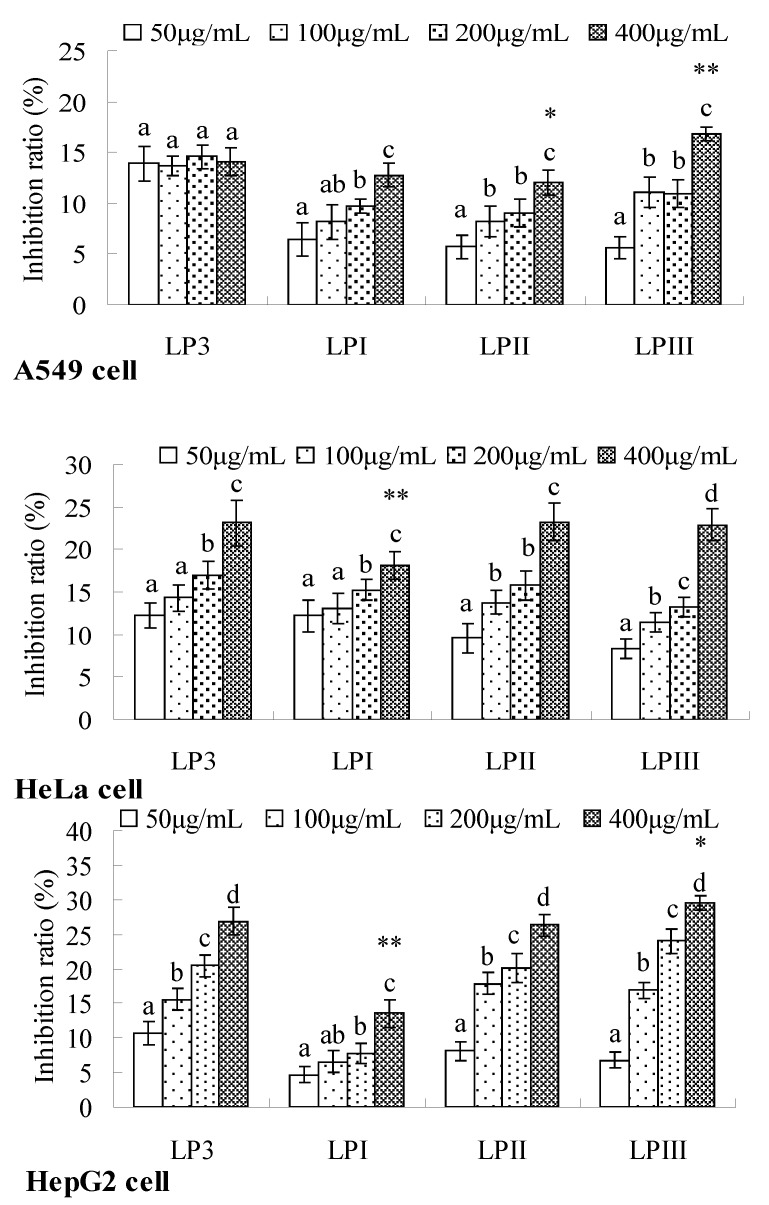
Inhibitory effects of longan polysaccharides on tumor cells *in vitro*. LPI–III were the fractions isolated from LP3. The cell proliferation was detected using a modified methylene blue method. Column expressed values were significantly different if marked with different letters (*p* < 0.05). At 400 µg/mL, the experimental value which showed significant difference from that of LP3 treated group was marked with * (*p* < 0.05) or ** (*p* < 0.01).

The anti-tumor mechanism of polysaccharides is usually proposed to be the immunostimulation of cell-mediated immune responses, but some polysaccharides, such as those from *Hedysarum polybotrys* Hand.-Mazz [38], *Chlorella pyrenoidosa* [39], *Anemone raddeana* [40] and *Angelica sinensis* (Oliv.) Diels [41], could directly inhibit the proliferation of tumor cell *in vitro*. As seen in Figure 6, the inhibitory effects of longan polysaccharides on A549, HeLa, and HepG2 cells generally strengthened with increasing dose in the range of 50–400 µg/mL, and the inhibition ratio of 400 µg/mL was significantly higher than those of other doses (*p* < 0.05). 

Especially, there were no significant differences among the A549 cell groups treated with various doses of LP3 (*p* > 0.05). The inhibition ratios of longan polysaccharides in A549 and HeLa cells respectively ranged from 5.6% to 16.8% and 8.3% to 23.2%, were comparable to those of polysaccharide from *Angelica sinensis* (Oliv.) Diels [41] but obviously weaker than those of *Chlorella pyrenoidosa* polysaccharides [39]. Longan polysaccharides had inhibition ratios ranging from 4.7% to 29.5% in HepG2 cell proliferation, showing a considerable anti-tumor activity compared with the polysaccharides from *Dendrobium nobile* Lindl [42], *Pleurotus tuber regium* [13] and rice bran [43].

At the dose of 400 µg/mL, the inhibitory effects of LPI on HeLa and HepG2 cells were significantly weaker than those of LP3 (*p* < 0.01), LPII possessed the lowest inhibition ratio in A549 cell proliferation, compared with other polysaccharides, and LPIII showed stronger inhibition on A549 and HepG2 cells than LP3, with significant differences at *p* < 0.01 and *p* < 0.05, respectively. The contributions of the factors related to the anti-tumor activity of polysaccharide were of the order of water solubility > chain conformation > *M*_w_ [13]. Longan polysaccharides exist as semi-flexible chains, in detail: LPI had both a compact sphere-like conformation and a highly-organized triple-helix structure [36], and LPII–V all existed as flexible chains. The formation of LPII–IV conformations might be related to the effects of sodium ion during the elution process and the negative charges they possess. The anti-tumor activity of LPIII was significantly stronger than that of LPI at 400 µg/mL (*p* < 0.01). Moreover, the immunomodulatory effects of LPI–IV on splenic lymphocytes and NK cells were ordered as LPIII > LPIV > LPII > LPI [36]. Redundant side chain branches hindered polysaccharides from forming a proper fold, which is important for cell receptor recognition, resulting in a weakening of the immunostimulatory response [44]. The potential structure-activity relationship could be indicated that the bioactive conformation for the anti-tumor and immunomodulatory activities of longan polysaccharides was a flexible chain, but not in a sphere-like conformation.

## 3. Experimental

### 3.1. Preparation of Longan Pulp Polysaccharide Fractions

Longan fruits (cv. Chu-liang) were provided by Pomology Research Institute of Guangdong Academy of Agricultural Sciences (Guangzhou, China). Four fractions (LPI–IV) were purified from crude longan polysaccharide LP3 by using a DEAE-52 cellulose column [19,20]. LPI was a neutral fraction, the others were all acidic fractions. Their total sugar content determinate by phenol-sulphuric acid method and expressed as glucose equivalents were 98.72%, 93.56%, 96.49% and 96.58%, respectively. The major monosaccharides in LP3 and LPI were both glucose and mannose (molar ratio, 2.2:1.0). The protein bound polysaccharides of LPII was mainly composed of glucose, mannose, arabinose, galactose and ribose at the molar ratio of 14.6:5.8:3.0:1.8:1.0. LPIII was mainly composed of arabinose, rhamnose, galactose and ribose at the molar ratio of 4.7:3.2:2.2:1.0. LPIV was mainly composed of xylose, ribose, mannose and glucose at the molar ratio of 7.8:5.0:2.3:1.0. The weight average molecular masses of LPI–IV were 1.459 × 10^4^, 6.834 × 10^4^, 1.074 × 10^5^ and 5.282 × 10^6^ g/mol, respectively [20].

### 3.2. Aqueous Solution Property Analysis

*Molecular size measurement*: The root-mean-square radii of gyration (<S^2^>_z_^1/2^) of LPI–IV were measured by size-exclusion chromatography combined with laser light scattering method [36]. Size-exclusion columns (Shodex SB-804 connected with Shodex SB-802) equipped with a pump (Waters 515 HPLC) were simultaneously connected with a multi-angle laser light photometer (λ = 633 nm, Wyatt-DAWN HELEOS-II, Wyatt Technology Co., Santa Barbara, CA, USA) and a differential refractive index detector (RI, Wyatt-Optilab rex). 200 µL sample aqueous solution in the concentration range of 3.0–5.0 mg/mL was injected. 0.1 mol/L sodium nitrate was used as isocratic mobile phase at the flow rate of 0.5 mL/min. A specific refractive index increments (dn/dc) value of the polysaccharides in distilled water, which was determined by OPTILAB DSP differential refractometer (Wyatt Technology Co.) at 633 nm and 25 °C, was 0.147 mL/g.

*Intrinsic viscosity measurement*: Intrinsic viscosities ([*η*]) of LPI–IV in water were measured at 25 ± 0.05 °C by using an Ubbelohde capillary viscometer (0.46 mm). The polysaccharide solutions in the concentration range of 0.8–1.2 mg/mL were prepared, and were measured after passing through a fritted glass Buchner funnel (15–40 µm) [45]. The data obtained were averages of at least three measurements. The kinetic energy correction was negligible. The Huggins equation was used to estimate the [*η*] value by extrapolation to infinite dilution as *η*_sp_/c = [*η*] + k’ [*η*]^2^c, where *η*_sp_/c was the reduced viscosity, c was the polysaccharide concentration, and k’ was a constant.

*Complex formation with Congo red*: The helical structure of polysaccharide was identified by characterizing Congo red-polysaccharide complex [36]. In brief, polysaccharide solution (2 mL, 0.5 mg/mL) was mixed with Congo red solution (2 mL, 50 µmol/L) in a tube, and NaOH solution (1 mL, final concentration: 0, 0.05, 0.10, 0.15, 0.20, 0.25, 0.30, 0.35, 0.40, 0.45 or 0.50 mol/L) was then added. Meanwhile, distilled water (2 mL), Congo red solution (2 mL) and NaOH solution (1 mL) were mixed as control. After 10 min at room temperature, the maximum absorption wavelength (λ_max_) of the mixture was scanned in the range of 400–600 nm.

*Observation with atomic force microscope*: The image of polysaccharide which was prepared onto freshly cleaved ruby muscovite mica substrate was obtained in air operating an atomic force microscope (Multimode Nanoscope III, Veeco Metrology, Santa Barbara, CA, USA) equipped with etched silicon tips (Veeco RTESP probes, 274–335 kHz and 20–80 N/m) in the tapping mode [36].

### 3.3. Anti-Tumor Activity Evaluation

Human lung adenocarcinoma A549 cells, human cervix carcinoma HeLa cells and human hepatoma HepG2 cells were kindly provided by Experiment Animal Center of Sun Yat-sen University (Guangzhou, China). The proliferation assay of tumor cell was detected by a modified methylene blue method [46]. Briefly, A549, HeLa and HepG2 cells were respectively adjusted to 4 × 10^4^, 2 × 10^4^, 1 × 10^4^ cell/mL by DMEM culture medium containing 10% fetal bovine serum (Gibco BRL, Grand Island, NY, USA). 100 µL/well of tumor cell was plated in 96-well culture plates and allowed to attach (6 h for A549 and HeLa; 4 h for HepG2) at 37 °C in 5% CO_2_. The media were aspirated. Polysaccharide dissolved in culture medium was then added in each well (volume, 100 µL; final concentrations, 50, 100, 200 or 400 µg/mL). After 72 h incubation at 37 °C in 5% CO_2_, the polysaccharide-containing media were removed and each well was gently rinsed twice with phosphate buffered saline (PBS). Cells were stained and fixed by adding 50 µL methylene blue solution (Hank’s balanced salt solution containing 1.25% glutaraldehyde and 0.6% methylene blue). After 1 h incubation at 37 °C in 5% CO_2_, wells were washed with distilled water. Plates were drained and air-dried. Each well was added in 100 µL elution solution composed of 50% ethanol, 49% PBS and 1% acetic acid for 15 min at room temperature. The absorbance at 570 nm (A_570_) was measured on a microplate reader (Thermo Labsytems, Helsinki, Finland). The inhibition ratio of the treated cells was calculated based on the following formula:

(1 − the A_570_ value for treated cells/the A_570_ value for untreated cells) × 100%

### 3.4. Statistical Analysis

The data were expressed as the mean ± SD of six replications. Significance of difference was evaluated by one-way ANOVA, followed by the Student-Newman-Keuls test or least significant difference test using SPSS 11.5 software.

## 4. Conclusions

Longan polysaccharides, in the *M*_w_ range of 1.459 × 10^4^–5.282 × 10^6^ g/mol, the <S^2^>_z_^1/2^ range of 43.2–77.3 nm and the [*η*] range of 9.945–452.1 mL/g, were confirmed to exist as semi-flexible chains. Crude longan polysaccharide LP3 and its fractions LPI–IV exhibited direct inhibitory effects on tumor cells *in vitro*, indicating the potential for their use in cancer prevention. The conformation contributing to the anti-tumor activities of longan polysaccharides could be their flexible chains.
